# Edaravone Protect against Retinal Damage in Streptozotocin-Induced Diabetic Mice

**DOI:** 10.1371/journal.pone.0099219

**Published:** 2014-06-04

**Authors:** Dongqing Yuan, Yidan Xu, Hui Hang, Xiaoyi Liu, Xi Chen, Ping Xie, Songtao Yuan, Weiwei Zhang, Xiaojun Lin, Qinghuai Liu

**Affiliations:** Department of Ophthalmology, The First Affiliated Hospital with Nanjing Medical University, Nanjing, China; Case Western Reserve University, United States of America

## Abstract

Edaravone (3-methyl-1-phenyl-2-pyrazolin-5-one), a free radical scavenger, is used for the clinical treatment of retinal injury. In this study, we investigated the protective effects of edaravone against diabetic retinal damage in the mouse. Diabetic retinopathy in the mouse was induced by injection of streptozotocin. Edaravone was given once-daily and was intraperitoneally (i.p.) treated at a dose of 3 mg/kg from streptozotocin injection to 4 weeks after onset of diabetes. Retinal ganglion cells (RGCs) damage was evaluated by recording the pattern electroretinogram (ERG). RGCs damage was also detected by Terminal deoxynucleotidyl transferase dUTP nick end labeling (TUNEL) staining, and the levels of reactive oxygen species (ROS) were determined fluorometrically. The expressions of phosporylated-ERK1/2, BDNF, and caspase-3 were determined by Western blot analysis. Retinal levels of ROS, phosphorylated ERK1/2, and cleaved caspase-3 were significantly increased, whereas the expression of BDNF was significantly decreased in the retinas of diabetic mice, compared to nondiabetic mice. Administration of edaravone significantly attenuated diabetes induced RGCs death, upregulation of ROS, ERK1/2 phosphorylation, and cleaved caspase-3 and downregulation of BDNF. These findings suggest that oxidative stress plays a pivotal role in diabetic retinal damage and that systemic administration of edaravone may slow the progression of retinal neuropathy induced by diabetes.

## Introduction

Diabetes produces a spectrum of retinal abnormalities that result in damage to the vasculature and neurons, and in severe cases, loss of vision itself. The pathogenesis of diabetic retinopathy remains to be elucidated, although reduction in hyperglycemia has been shown to exert positive effects on the development and progression of diabetic retinopathy [1]. Nevertheless, achievement and maintenance of glycemic control has been difficult or impossible in many patients, therefore effective therapies are needed to inhibit the retinopathy. Recent studies have shown that the retina has a high content of polyunsaturated fatty acids and has the highest oxygen uptake and glucose oxidation relative to any other tissue. This phenomenon renders retina more susceptible to oxidative stress [2]. In addition, oxidative stress induced caspase-independent apoptosis of retinal ganglion cells (RGC) in vitro [3]. Oxidative stress, which may occur because of an imbalance between the production and removal of reactive oxygen species (ROS), is considered to be a critical mediator in RGC injury of various etiologies and has been implicated in RGC death in the diabetic retinopathy.

Edaravone is a potent free radical scavenger and has been prescribed clinically in Japan since 2001 for the treatment of acute brain infarction [4]. It also has protective effects against cerebral ischemia and reperfusion injuries in a variety of experimental animal models [5]. Previous study has shown the protective effects of intraperitoneal or intravitreous edaravone treatments against light-induced retinal damage in mice [6]. These protective effects of edaravone are thought to be attributable to its scavenging of ROS. However, it remains unclear whether edaravone protects against RGC death with diabetic retinopathy through ROS-scavenging effects.

In the present study, our aim was to investigate the potential protective effects of edaravone against RGC death with diabetic retinopathy.

## Materials and Methods

### 2.1 Animals

Six-week-old male C57BL/6 mice were obtained from Nanjing Medical University (Nanjing, China). Mice were maintained in temperature-controlled clean racks with a 12-h light/dark cycle. This study was carried out in strict accordance with the recommendations in the guide for the care and use of animals of the Association for Research in Vision and Ophthalmology (ARVO). The protocol was approved by the Committee on the Ethics of Animal Experiment of the First Affiliated Hospital with Nanjing Medical University (Permit Number: 22-005029). All reasonable efforts were made to minimize suffering.

### 2.2 Generation of Diabetic Mouse Model

Diabetic mice were generated by a single intraperitoneal injection of streptozotocin (150 mg/kg body weight; Sigma). Streptozotocin was freshly prepared in 100 mM citrate buffer (pH 4.5). After injection, mice were supplied with 10% sucrose overnight to prevent sudden hypoglycaemic shock. Sufficient hyperglycaemia was observed 2 days after injection, as determined by measuring blood glucose using the Accu-Check Active blood glucose monitor (Roche Diagnostics, Germany). After 1 week, mice with non-fasting blood glucose levels >16 mM, polyuria, and glucosuria were defined as diabetic and used for the experiments.

### 2.3 Treatment with Edaravone

Mice were separated into three groups. Two groups received streptozotocin injection for diabetic model groups. For Edaravone group, Edaravone was given once-daily and was intraperitoneally (i.p.) treated at a dose of 3 mg/kg from streptozotocin injection to 4 weeks after onset of diabetes. For Vehicle group, an identical volume of saline was given intraperitoneally. One group was as a normal group without any treatment. The dose of Edaravone was selected based on previous studies that had demonstrated the effectiveness of the drug [7]–[9]. Edaravone (Mitsubishi Pharma Corporation, Tokyo, Japan) was dissolved in dimethyl sulfoxide (DMSO) and was diluted with physiologic saline to a concentration of 3 mg/mL.

### 2.4 Electroretinograms

Electroretinograms (ERGs) were recorded at 4 weeks after onset of diabetes by streptozotocin (Roland Consult, Brandenburg, Germany). Mice were initially anesthetized with an intraperitoneal injection of a mixture of ketamine (120 mg/kg; Daiichi-Sankyo, Tokyo, Japan) and xylazine (6 mg/kg; Bayer Health Care, Tokyo, Japan). Pattern ERGs were recorded in response to contrast-reversal of horizontal black and white square wave bar gratings with a mean luminance of 50 photopic (ph) cd/m2, and more appropriate for mouse M-cones, 45 scotopic (sc) cd/m2 (calibrated using model IL 1700 with a scotopic filter, International Light, Peabody MA, USA). The standard grating contrast was 90%, with a 1 Hz reversal rate, and spatial frequency of 0.05 cy/deg. For a subset of animals, contrasts between 50 and 90% were used to ensure that the amplitude of both positive (P_50_) and negative (N_95_) portions of the response were optimized. The stimulus display contained four full cycles of the grating. The center of visual stimulus was aligned with the projection of the pupil. A typical protocol consisted of a series of 6 consecutive responses to 200 contrast reversals each, and then the 1200 reversals were averaged [10]. Body temperature was monitored and maintained at 37°C with a thermostatically controlled electrically heated blanket (CWE, Inc., Ardmore, PA, USA). The amplitude of P_50_ was measured from baseline at the positive peak of P_50_. The full PERG amplitude, in most cases was measured from the positive peak of P_50_ to the negative trough of N_95_, and referred to as N_95_.

### 2.5 TUNEL Staining

Terminal deoxynucleotidyl transferase dUTP nick-end labeling (TUNEL) staining was performed according to the manufacturer’s protocols (In Situ Cell Death Detection Kit; Roche Biochemicals, Mannheim, Germany) to detect diabetic RGC death. The mice were anesthetized with 80 mg/kg pentobarbital sodium administered intraperitoneally 4 weeks after onset of diabetes. Their eyes were enucleated, fixed overnight in 4% paraformaldehyde, immersed for 2 days in 25% sucrose with PBS, and were then embedded in a supporting medium for frozen-tissue specimens (OCT compound; Tissue-Tek, Naperville, IL). Retinal sections 10 µm thick were cut on a cryostat at −25°C and stored at −80°C until staining. After two washes with PBS, the sections were incubated with terminal deoxyribonucleotidyl transferase enzyme at 37°C for 1 hour and were then washed three times in PBS for 1 minute at room temperature. After that, the sections were incubated with an anti-fluorescein antibody-peroxidase conjugate at room temperature in a humidified chamber for 30 minutes and were then developed using DAB tetrahydrochloride peroxidase substrate. Light microscope images were photographed, and labeled cell counts in the RGC layer through the optic disc were obtained in retina. The number of TUNEL-positive cells was averaged for the whole RGC layer.

### 2.6 Western Blot Analysis

Retinas were homogenized in a western lysis buffer (30 mM Tris-HCl, pH 7.4, 250 mM Na3VO4, 5 mM EDTA, 250 mM sucrose, 1% Triton X-100 with protease inhibitor and phosphatase inhibitor cocktail). The lysate was centrifuged at 14,000×g for 10 min at 4°C, and the supernatant was collected. Protein content was assayed by DC protein assay (Bio-Rad Laboratories, Hercules, CA, USA). The tissue lysates containing 40–50 µg protein were separated on 8–15% SDS-polyacrylamide gels and were transferred onto nitrocellulose membranes. The blots were blocked with 5% nonfat milk in TBST (20 mM Tris-HCl, pH 7.6, 136 mM NaCl, and 0.1% Tween-20).

For detection of p-ERK1/2, brain-derived neurotrophic factor (BDNF), and cleaved caspase-3, the membrane was incubated overnight at 4°C with rabbit polyclonal anti-ERK1/2 (1∶300, Cat. no. ab17942, Abcam), mouse monoclonal anti-phosphorylated ERK1/2 (1∶300, Cat. no. ab50011, Abcam), mouse monoclonal anti-BDNF (1∶500, Cat. no. SC-65513, Santa Cruz Biotechnology), and rabbit monoclonal anti-caspase-3 (1∶300, Cat. no. MAB835, R&D Systems). After overnight incubation with primary antibodies, the membranes were washed four times with TBS-T (5 min each). For p-ERK1/2, and BDNF, the membrane was incubated at room temperature for 1.5 h with anti-mouse secondary horseradish peroxidase-conjugated antibody (1∶2000, SC-2005, Santa Cruz Biotechnology), and for ERK1/2 and cleaved caspase-3, with anti-rabbit secondary horseradish peroxidase-conjugated antibody (1∶2000, SC-2004, Santa Cruz Biotechnology). After incubations with secondary antibodies, membranes were washed four times with TBS-T (5 min each) and the immunoreactivity of bands was visualized on a high-performance chemiluminescence machine (G: Box Chemi-XX8 from Syngene, Synoptic Ltd. Cambridge, UK) by using enhanced chemiluminescence plus Luminol (SC-2048, Santa Cruz Biotechnology) and quantified by densitometric analysis using image processing and analysis in GeneTools (Syngene by Synoptic Ltd. Cambridge, UK). As a control, the blots were stripped and detected with a mouse monoclonal anti-β-actin (1∶2000, SC-47778, Santa Cruz Biotechnology), antibody. All data from the three independent experiments were expressed as a ratio to OD.

### 2.7 Reactive Oxygen Species Measurements

Reactive Oxygen Species (ROS) generation was measured in retinal tissue homogenates using a 2′,7′-dichlorofluorescein-diacetate (DCFHDA) [11]. DCFHDA, a nonfluorescent dye, is cleaved by esterase activity to yield DCFH, which is subsequently oxidized by a variety of ROS to form dichlorofluorescein (DCF), which is fluorescent. Retinas were homogenized in PBS in presence of protease inhibitor using a glass homogenizer. Samples containing 20 µg proteins diluted in PBS were incubated with 5 µM DCFHDA (Invitrogen, CA, USA) in the dark for 15 min. Fluorescence was measured using a spectraMax Gemini-XPS (Molecular Devices, CA, USA) every 15 min for 1 h with excitation and emission wavelengths of 488 nm and 525 nm.

### 2.8 Statistical Analysis

Each measurement was made in duplicate, and the assay was repeated three or more times. Data are expressed as mean ± SD and experimental groups were compared using the nonparametric Kruskal-Wallis test followed by the Mann-Whitney test for multiple-group comparison. A value <0.05 indicated statistical significance. SPSS version 13.0 (SPSS Inc., Chicago, IL) was used for the statistical analyses.

## Results

### 3.1 Electroretinograms and TUNEL-Positive Cells

**Figure 1:**
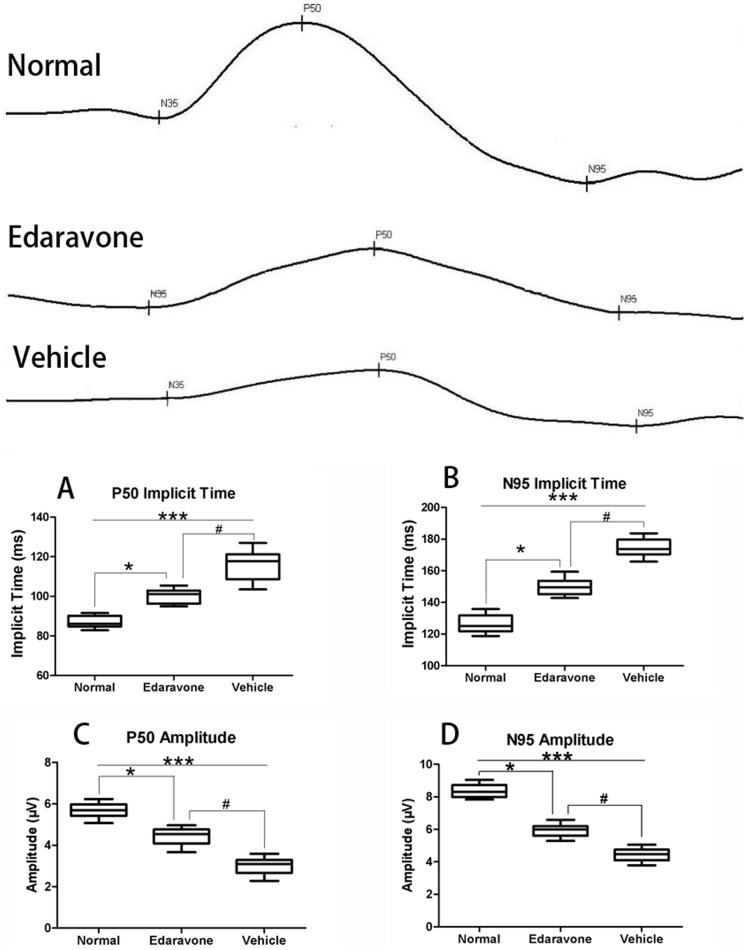
Pattern ERG responses in the Normal, Edaravone and Vehicle groups. (A) Average contrast implicit time of P50 response in the three groups. (B) Average contrast implicit time of N95 response in the three groups. (C) Average contrast amplitude of P50 response in the three groups. (D) Average contrast amplitude of N95 response in the three groups. The nonparametric Kruskal-Wallis test followed by the Mann-Whitney test was used for multiple-group comparison. Values are the means ± SD of three independent experiments.*P<0.05, ***P<0.001 vs. Normal, ^#^P<0.05 vs. Edaravone.

Figure 1 shows the measurement of the pattern ERG implicit time and amplitudes at 7 days after treatment in the mouse retina. In the normal mouse retinas, the mean amplitude of P_50_ was about 5.69±0.37 µV; the implicit time of P_50_ was about 86.97±3.02ms; the mean amplitude of N_95_ was about 8.35±0.40 µV; and the implicit time of N_95_ was about 126.41±5.57ms. In the Edaravone group mouse retinas, the four results were changed into 4.44±0.41 µV, 100.18±3.60ms, 5.93±0.40 µV, and 149.78±5.09ms, respectively. In the Vehicle group mouse retinas, the four results were changed into 3.02±0.42 µV, 115.58±7.59ms, 4.43±0.42 µV, and 174.46±5.41ms, respectively. The implicit time and amplitude of the P_50_ wave of Vehicle and Edaravone groups were significantly delayed and reduced (P<0.0001 and P<0.01, respectively); the implicit time and amplitude of the N_95_ wave were significantly delayed and reduced (P<0.0001 and P<0.01, respectively) compared with non-diabetic control group. However, there was also significant difference between the pattern electroretinogram (ERG) of Vehicle group and Edaravone group (P<0.01 and P<0.01, respectively).

**Figure 2:**
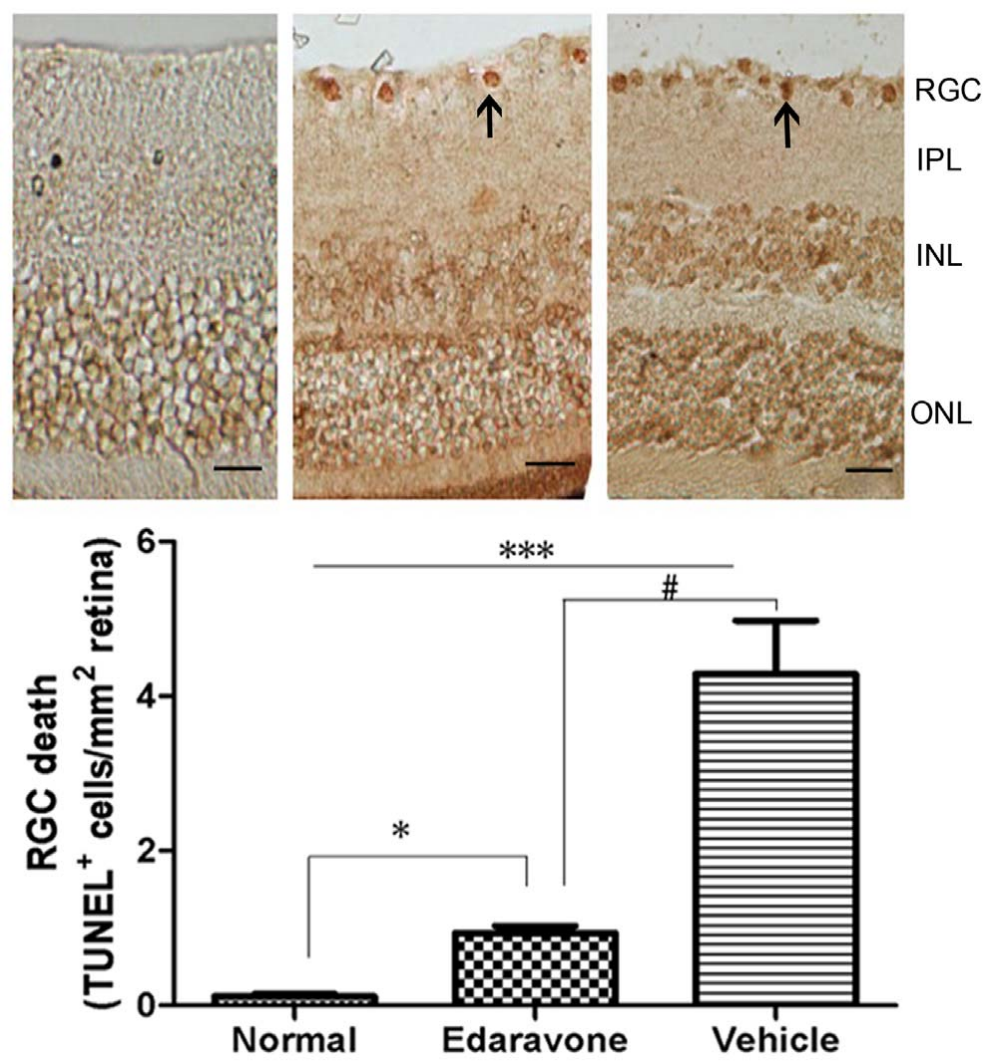
Eeffects of edaravone on RGC death with TUNEL staining. Quantification of TUNEL (+) cells showed significantly decreased TUNEL (+) cells/mm^2^ with treatment of edaravone, compared with the streptozotocin-treated group. Data are expressed as the mean±SD. *P<0.05, ***P<0.001 vs. Normal, ^#^P<0.05 vs. Edaravone. Original magnification: ×10; scale bar, 100 µm.

**Figure 3:**
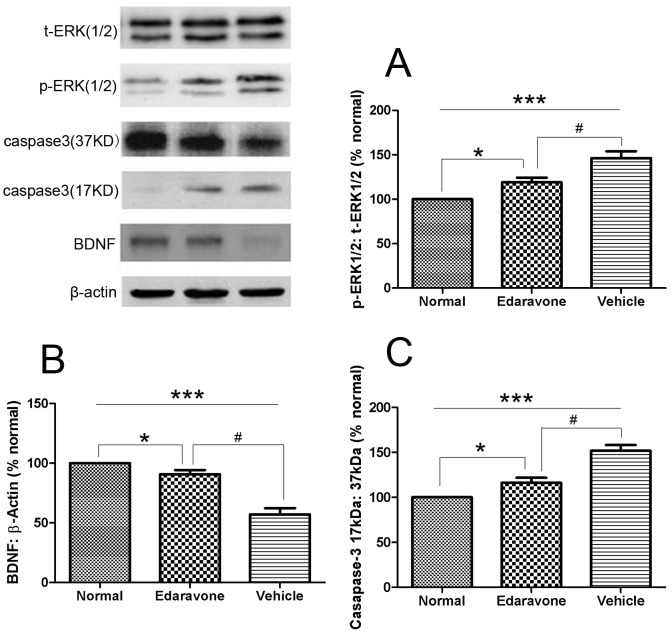
Eeffects of edaravone on retinal ERK1/2 activation, BDNF expression and cleavage of caspase-3 in diabetic retinopathy. (A) Data are expressed as percentage change in phosphorylated ERK1/2 (p-ERK1/2) over total ERK1/2 (t-ERK1/2) (B) The histogram represents the mean band intensity of BDNF adjusted to the intensity of β-actin in the same sample. (C) The ratio of active caspase-3 (17 kDa) and pro-caspase-3 (37 kDa) was calculated in control and diabetic mice maintained with and without Edaravone treatment. Measurements were made in duplicate in six to eight mice in each group. Western blots are representative of three different experiments. Results are expressed as mean±SD. Values obtained from Normal group are considered as 100%. *P<0.05, ***P<0.001 vs. Normal, ^#^P<0.05 vs. Edaravone.

Maintenance of functional cell mass is critical for signal transduction. Dysfunction induced by the decreased population of cells is regarded as an important factor in the pathogenesis of various metabolic diseases [12], [13]. As shown in Figure 1, we investigated the function of RGCs which had significantly reduced in retina of diabetes mice, compared with normal mice, and edaravone reversed such effects. To determine the effects of edaravone on RGCs, the apoptosis was determined using tunel assays. The detection of DNA damage by tunel staining indicated that diabetes could induce the apoptosis of RGCs and edaravone decreased the apoptosis of RGCs in retina (Figure 2). Cleaved caspase-3, the apoptosis executer enzyme, was significantly upregulated in diabetic retinas compared to nondiabetic controls. Cleaved caspase-3 levels in the retinas of diabetic mice were increased by about 51% compared to non-diabetic controls, but in edaravone group was increased by about 16% (Figure 3C). These results indicated that treatment of edaravone could reduce the apoptosis of RGCs in mice with diabetes.

### 3.2 Effect of Diabetes on Retinal Expression of Mediators and Markers of Neurodegeneration

Western blot analysis demonstrated significant upregulation of ERK1/2 phosphorylation in diabetic retinas compared to nondiabetic retinas. The phosphorylation of ERK1/2 protein in the retinas of diabetic mouse was upregulated by about 46% as compared to the retinas of normal group, but in edaravone group was upregulated by about 19% (Figure 3A). The neurotrophin brain-derived neurotrophic factor (BDNF) was significantly downregulated in diabetic retinas compared to nondiabetic controls. BDNF expression in the retinas of diabetic mice was decreased by about 43% compared to nondiabetic controls, but in edaravone group was only 10% (Figure 3B). Taken together, it suggested that edaravone had a strong anti-apoptotic effect by inhibition of daibetes-induced oxidative stress in RGCs of retinas.

### 3.3 Effect of Diabetes on Retinal ROS Generation

**Figure 4:**
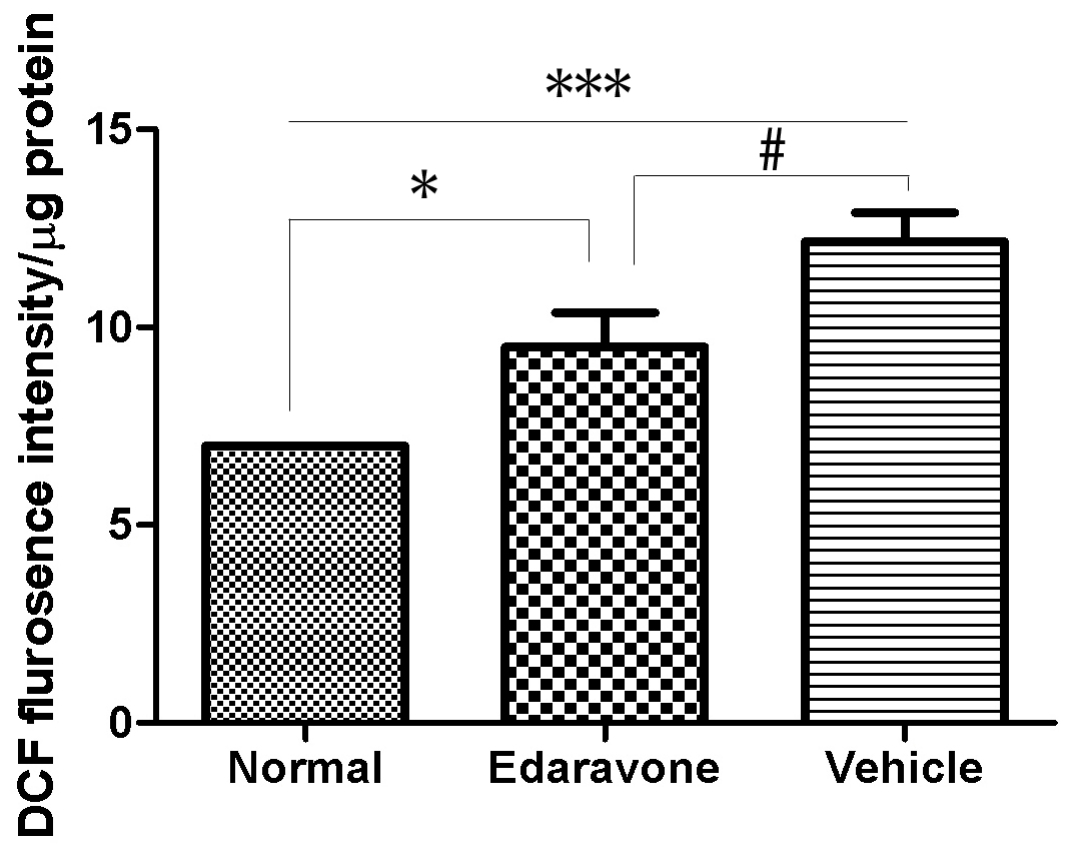
Effect of Edaravone on retinal reactive oxygen species (ROS) levels in diabetes. Freshly prepared retinal homogenates were incubated with DCHFDA (5 µM) for 15 min. An equal amount of protein was used to quantitate 2′,7′-dichlorofluorescein fluorescence. Data are expressed as percent normal control and mean±SD from retina in each group. *P<0.05, ***P<0.001 vs. Normal, ^#^P<0.05 vs. Edaravone.

Spectrofluorometric analysis demonstrated significant upregulation of ROS generation in diabetic retinas compared to nondiabetic retinas. ROS production in retinas was determined by DCFH-DA probe. The levels of DCF fluorescence intensity in the retina of diabetic mice were increased by about 50% as compared to control group. And in the treatment with edaravone group the ROS production was significantly reduced by about 25% as compared to Vehicle group (Figure 4).

## Discussion

In the present study, to investigate the role of oxidative stress in retinal damage after diabetes, we focused on the effects of edaravone, a free radical scavenger, against retinal damage by diabetes-induced retinopathy. In our rodent model of diabetic retinal neuropathy, we found evidence of a substantial increase in oxidative stress. In the diabetic retinas, RGCs were significantly reduced, but ROS, p-ERK1/2, and cleaved caspase-3 levels were significantly increased at the same time. On the other hand, diabetes induced significant downregulation of the neurotrophin BDNF. Treatment with the free radical scavenger edaravone, significantly reduced the expression of oxidative stress markers, along with a decrease in TUNEL-positive cells, which demonstrated remarkable neuroprotective effects against diabetes-induced RGC degeneration in mice.

Diabetic C57BL/6 mice exhibited progressive loss of RGCs in the ganglion cell layer during diabetes [14]. We found that by four weeks after onset of diabetes, the number of RGCs was reduced by approximately 40% of the normal number and a gradual decrease in the number of RGCs was demonstrated. Therefore, there is general agreement that all mice strains have shown RGC loss or damage in diabetes [7], [15]–[23]. It is well-known that oxidative stress plays a crucial role in the development of diabetes mellitus [24], [25]. However, the exact mechanism of its development remains elusive. It has been suggested that ROS generation is activated in the diabetic retinopathy, leading to the initial production of superoxide (O2•−) and nitric oxide (•NO) radicals. These ROS then react and metabolize to form stronger oxidants in the form of peroxynitrite (ONOO−), hydroxyl (•OH), carbonate (CO3•−), and nitrogen dioxide (•NO2) radicals [24], [25], which in turn react with proteins, lipids, sugars, and nucleotides and impair the normal physiological function of cells. Our findings suggest that administration of edaravone significantly attenuated diabetes induced ROS generation.

Enhanced ROS level causes reduced levels of BDNF, a protein belonging to the neurotrophin family. BDNF is expressed in RGCs and Müller cells [26] and is important for the survival of retinal ganglion cells [27]. BDNF have been known to play crucial roles in promoting neuronal survival and preventing cell death. Several previous studies confirmed that edaravone could elevate BDNF and Bcl-2 expression levels using in vitro and in vivo ischemic models [28], [29]. Our observations demonstrated that edaravone treatment largely promoted the expression levels of BDNF diabetic retinas. Furthermore, edaravone application blockade strongly depressed the increase of caspase-3 activity in response to diabetes-induced neurotoxicity. These results indicated that up-regulation of BDNF is likely to participate in edaravone neuroprotection, which is consistent with previous reports.

Edaravone has been previously reported to protect the apoptosis of photoreceptor cells has been related to the activation of both intrinsic and extrinsic apoptotic pathways in experimental retinal detachment rats [30]. Shimazaki et al. demonstrated that edaravone protects against light-induced photoreceptor degeneration in the mouse retina [6], [8]. However, the machnism of edaravone alleviates diabetes-induced neuronal injury was unclear. MAPKs are stress-related kinases, and members of the MAPKs subfamily (JNK, p38, and ERK1/2) have been implicated in neuronal injury and diseases [31]. ERK1/2 is activated by oxidative stress, mitogens, and survival factors, and that regulates cell proliferation and differentiation [32]. Although the exact roles played by ERK1/2 were not clear, ERK1/2 may act in some cases to promote cell survival while also participating in neuronal cell death and neurodegeneration. Kawasaki et al. provided evidence that edaravone protected rat astrocytes against nitric oxide-induced cytotoxicity by inhibiting ERK1/2 activation [33]. Under our experimental conditions, edaravone pretreatment following streptozotocin injection greatly diminished p-ERK1/2 expression, indicating that ERK1/2 activation participates in edaravone neuroprotection during diabetic retinopathy. These results suggest that ERK1/2 acts downstream of edaravone and plays a vital role in the neuroprotection of edaravone under diabetic neurodegeneration.

Our study also has several limitations. In diabetes, retinal neuropathy is associated with enhanced oxidative stress resulting from excess generation of ROS that often leads to retinal microvascular cell death [34], [35]. We found that the vascular endothelial cells and pericytes were reduced in diabetes models and protective effect of edaravone on them in the retina, which were similar to the previous research. In the further study, we would add that results to support our conclusions. In addition, as the number of RGCs was gradually decreased, we would maintain animals for additional months after onset of diabetes to see further effects of edaravone in diabetic retinas. Future experimental studies aimed at analysing the long-term effect of edaravone administration in diabetic retinopathy are anticipated.

In conclusion, our study demonstrated that administration of edaravone might be a novel approach for the prevention of diabetes retinopathy. Our results provided a new insight into potential beneficial effects of antioxidants in the treatment of retinopathy in diabetic patients.
